# Prevalence of Eating Disorders in Adults with Celiac Disease

**DOI:** 10.1155/2013/491657

**Published:** 2013-12-04

**Authors:** V. Passananti, M. Siniscalchi, F. Zingone, C. Bucci, R. Tortora, P. Iovino, C. Ciacci

**Affiliations:** ^1^Department of Clinical and Experimental Medicine, University Federico II of Naples, Via S. Pansini, 80131 Naples, Italy; ^2^Department of Medicine and Surgery, University of Salerno, Baronissi Campus, Via S. Allende, 84081 Baronissi, Salerno, Italy

## Abstract

*Background*. Symptoms of celiac disease negatively impact social activities and emotional state. Aim was to investigate the prevalence of altered eating behaviour in celiac patients. *Methods*. Celiac patients and controls completed a dietary interview and the Binge Eating Staircases, Eating Disorder Inventory (EDI-2), Eating Attitudes Test, Zung Self-Rating Depression Scale, State Trait Anxiety Inventory Forma Y (STAI-Y1 and STAI-Y2), and Symptom Check List (SCL-90). *Results*. One hundred celiac adults and 100 controls were not statistically different for gender, age, and physical activity. STAI-Y1 and STAI-Y2, Somatization, Interpersonal, Sensitivity, and Anxiety scores of the SLC-90 were higher in CD patients than controls. EDI-2 was different in pulse thinness, social insecurity, perfectionism, inadequacy, ascetisms, and interpersonal diffidence between CD and HC women, whilst only in interceptive awareness between CD and HC men. A higher EAT-26 score was associated with the CD group dependently with gastrointestinal symptoms. The EAT26 demonstrated association between indices of diet-related disorders in both CD and the feminine gender after controlling for anxiety and depression. *Conclusion*. CD itself and not gastrointestinal related symptoms or psychological factors may contribute pathological eating behavior in celiac adults. Eating disorders appear to be more frequent in young celiac women than in CD men and in HC.

## 1. Introduction

Celiac disease (CD) is a chronic inflammatory immune-mediated disease. It is a common condition with a prevalence in the western world of about 1 : 100 [1, 2]. Characterized by a clinical heterogeneity, CD presents with a large spectrum of gastrointestinal and extra-intestinal symptoms and it may be diagnosed at any age [3–9]. CD patients also frequently experience somatisation, depression, and anxiety before diagnosis [10–12]. These psychological conditions are in general, associated with changes in appetite, and weight [13, 14]. A respectable body of data has demonstrated that an association exists between emotional dysfunction and eating disorders [13]. Only few studies, which utilized small sample sizes, though have explored emotional dysfunction and eating disorders in celiac disease [15, 16].

The present case-control study was designed to evaluate the prevalence of behavior suggestive of eating disturbances in untreated CD adult patients and to investigate a possible relationship between emotional-psychological factors and the presence of eating disorders.

## 2. Methods 

### 2.1. Study Population

Newly diagnosed CD adults were recruited from outpatient clinics devoted to food intolerances and celiac disease at the Federico II University of Naples and University of Salerno. All patients gave their written informed consent and the study protocol was approved by the Ethic Committee of the University Federico II of Naples (protocol: *Diagnosis and Follow-up of Celiac Disease in Adults*).

Patients' enrolment lasted from January 2011 to January 2012 and involved all newly diagnosed patients with CD meeting the following inclusion/exclusion criteria.

*Inclusion Criteria*. Caucasian adults all from the Southern Italian region Campania; age from 18 to 60 years; gastrointestinal symptoms at diagnosis (bloating, abdominal pain, diarrhea); no previous treatment with gluten-free diet; diagnosis of celiac disease according to Corazza-Villanacci histological classification [17] on well-performed biopsies [18], and presence of positive anti-transglutaminase antibodies and anti-endomysium antibodies [19].

*Exclusion Criteria*. IgA deficiency, the use of oral contraceptives, oral corticosteroid treatment, hormone replacement therapy, previous diagnosis of eating disorders, presence of other gastrointestinal diseases, pregnancy, diabetes, major psychiatric disorder, physical impairment limiting physical activity, and drug and alcohol abuse. Twenty-six patients were excluded because of presence of one or more exclusion criteria. We enrolled 100 CD patients at diagnosis. One hundred healthy individuals (HC) were enrolled from friends of CD patients and hospital staff and used as controls in comparison with CD patients. Inclusion criteria for HC were as follows. Caucasian adults from the Southern Italian region Campania; age from 18 to 60 years; no previous treatment for or diagnosis of celiac disease; negative serum anti-transglutaminase antibodies, and no IgA deficiency. Exclusion criteria were similar to that of CD patients with the addition of the genetic relationship to a CD patient.

For all participants we collected clinical and anthropomorphic data (BMI = weight_kg_/height_m_^2^). The clinical history included details related to weight reduction/gain, presence of gastrointestinal symptoms (diarrhea, constipation, abdominal pain, bloating, and vomiting), physical activity, and alcohol intake.

### 2.2. Data Sources

A psychologist (MS) performed a structured psychological assessment and administered the following questionnaires.

1. *EPIC Food Frequency Questionnaire*: participants are shown a photographic atlas of 16 food groups. Currently used in epidemiologic studies inSouthern Italy, these pictures depict typical Italian meals in order to help evaluate serving size and eating habits [20]. The food choices of subjects are “measured” with objective criteria helping to recreate food consumption. Although the atlas consists of 64 food/drink tables, patients in this study were only presented with the most frequently used meals in Southern Italy. In addition, we recorded the amount and frequency of consumption of carbonated soft drinks, chocolate and alcohol.
2. *Binge Eating Staircases (BES)*: BES measures the behavioral aspects of binge eating, as well as the feelings and thoughts associated with such behavior [21]. Originally created to investigate binge-eating behavior in obese patients, the scale has been also validated for nonobese patients and is composed of 16 multiple choice items. Based on the BES total score, individuals can be categorized into three groups according to established cut scores of binge eating severity [22]. A frequent convention is to use the BES as a screening measure to classify all participants with scores greater than or equal to 17 as “binge eaters” [23].
3. *Eating Attitudes Test (EAT-26)*: EAT-26 is a questionnaire with 26 items [24]. It investigates diet-related disorders, bulimia, and anxiety related to food. More specifically, EAT-26 highlights the perception of one's own weightand physical appearance. The EAT-26 test is a standardized and validated screening tool that can be considered a valuable aid in the diagnosis of eating disorders cross-culturally. Each question is scored on a scale of 0 to 3 (based on the replies: always, usually, often, sometimes, rarely, and never). A total score is obtained by adding the scores of each item. Possible scores on the EAT-26 range from 0 to 78. A score of 20 or above indicates that a person may have an eating disorder and is recommended for further evaluation by a mental health professional.
4. *Eating Disorder Inventory (EDI-2)* [25]: EDI-2 is a self-assessment instrument of the symptoms associated with anorexia nervosa and bulimia nervosa. In particular, it can be used to identify patients with “masked” eating disorders. The test can also detect and accurately measure psychological aspects or symptoms relevant to the treatment of eating disorders. The EDI-2 consists of 91 items and 11 scales and measures the following constructs: pulse thinness, bulimia, dissatisfaction for own body, interpersonal diffidence, perfectionism, inadequacy, interceptive awareness, fear of maturity, asceticism, impulsivity, and social insecurity. Each item is scored from 1 to 6: “always,” “usually,” “often” or “sometimes,” “rarely,” and “never.” We used the Italian version of EDI-2.
5. *Italian Version of the Original Zung Self-Rating Depression Scale (M-SDS)* [26, 27]: M-SDS investigates the presence of depressive symptoms. The M-SDS contains 20 items. In the M-SDS, a score of 44 is the cut-off value for pathological depression.
6. *STAI-Y1 and STAI-Y2*: are these scales which aim to measure the presence and grade of anxiety [28]. The questionnaires are each 20 items, with responses related to terms of intensity (from “almost never” to “almost always”). The items are grouped into two axes, which permit a distinction between existing anxiety (STAI-Y1) and predisposition to an anxious reaction as a personality characteristic (STAI-Y2). A score ≥40 is the cut-off value for both scales.
7. *Symptom Check List (SCL-90)*: SCL-90 is a self-administered questionnaire, consisting of 90 items. SCL-90 assesses a wide range of psychological and psychopathological symptoms by measuring internalizing (depression, somatization, and anxiety) and externalizing symptoms (aggression, hostility, and impulsivity). The items are grouped into 10 clusters (Somatization (SOM), Obsessive-compulsive (OC), Interpersonal Sensitivity (INT), Depression, (DEP) Anxiety (ANX), anger-hostility (HOS), phobic anxiety (PHOB), psychoticism (PSY), paranoid ideation (PAR), and sleep disturbances (SLEEP)). For each item, the patient responds using a scale of severity from 0 to 4, in reference to the last week [29].

### 2.3. Statistical Analysis

Percent frequencies and means with respective standard deviations were calculated for sample descriptive statistics

Unpaired Student's *t*-test was used to compare continuous data and *χ* ^2^ for categorical data. Univariate and multivariate regression analyses were performed as appropriate. The statistical program used was the Statistical Package for the Social Sciences (SPSS) for Windows, version 12.0. Statistical significance was accepted as *P* < 0.05.

## 3. Results

### 3.1. Study Population

All eligible CD patients (*n* = 100) and HC (*n* = 100) accepted to undergo psychological assessment and administration of questionnaires.  Table 1 depicts basic characteristics of the study population. CD patients were not significantly different from HC in regard to gender and age. Those in the CD group had a significantly lower BMI as compared to healthy controls. The percentage of individuals reporting moderate to high level of physical activity and percentage of subjects reporting some alcohol intake were not significantly different between the two groups (*P* = 0.7 and 1.0, resp.). As expected, CD patients had a significantly higher prevalence of gastrointestinal symptoms compared to that of HC's (Table 1).

### 3.2. Eating Behaviors 

#### 3.2.1. EPIC Food Frequency Questionnaire

The photographic atlas for assessing quality and quantity of food intake showed that fish and meat meal portions were similar between both groups. The overall carbohydrate intake was greater in CD patients compared to HC and this difference was statistically significant (*P* < 0.05). Specifically, the consumption of pasta and bread was approximately 30% higher in CD than in HC. Although the consumption of fruits and vegetables was slightly lower in CD than in HC, this difference was not statistically significant.

#### 3.2.2. Binge Eating Staircases

The percentage of pathological BES scores was similar in CD and HC groups (6% versus 0%; *P* = 0.09). In men BES pathological scores of CD compared with HC were statistical significant different (6% versus 0% *P* = 0.04). Zero women in the CD or HC groups had pathological scores for BES.

#### 3.2.3. Eating Attitudes Test (EAT-26)

A significantly higher EAT-26 score was found in the CD group compared to HC but was not independently associated with gastrointestinal symptoms (regression coefficient, *B* = 3.7, SE 1.7, *P* = 0.03). The percentage of pathological EAT-26 scores is significantly different between CD and HC groups (16% versus 4%; *P* = 0.01).

#### 3.2.4. Eating Disorder Inventory (EDI-2)

Table 2 profiles CD and HC groups according to EDI-2 scores in women and men. The EDI-2 questionnaire was originally validated only for young adults (16 to 26 years). Therefore, in order to avoid any age-related bias, analysis was repeated for CD and HC subgroups (n.76) with ages 18 to 26 years. Results were comparable to what was found in the entire cohort (data not shown). Compared to HC, CD women had significant differences in several items such as pulse thinness, social insecurity, perfectionism, inadequacy, ascetisms, and interpersonal diffidence compared to HC women, whilst CD men were different from HC men only for interceptive awareness. Eleven CD patients and 1 HC scored more than 20 items of the EDI-2 questionnaire. They were further interviewed (MS) and a clear eating disorder diagnosis was confirmed only in the 11 CD patients.

#### 3.2.5. M-SDS, STAY-1 and STAY-2, and SLC-90

**Figure 1:**
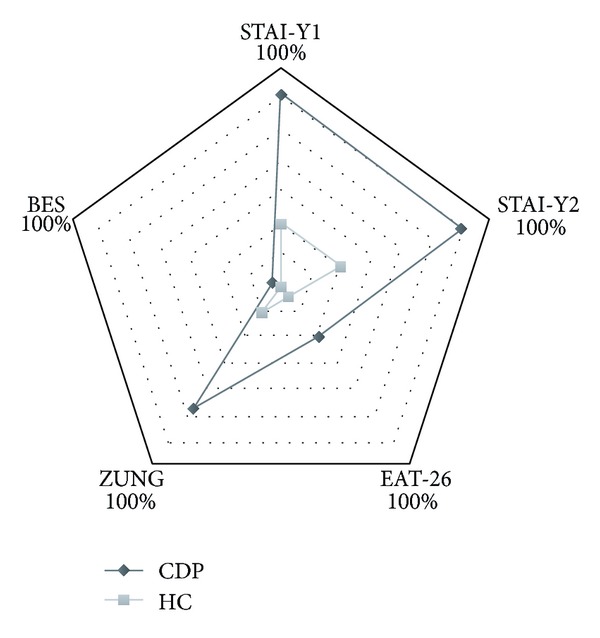
Graphic visualization of percentages of pathological scores of BES, M-SDS, EAT-26, STAY1, and STAY2 scales in both groups.

Figure 1 is a representation diagram that includes the results (expressed as percentage of pathological scores) of M-SDS, EAT-26, STAY1 and STAY2, and BES. Thirty-nine percent of CD patients and 6% of HC reported a pathological M-SDS (*χ* ^2^ 29.362, *P* < 0.001). STAY1 and STAY2 were also statistically significantly different between CD and HC groups (59% versus 18%, *χ* ^2^ 0.787, *P* < 0.001 and 61.4% versus 18.4%, *χ* ^2^ 33.908, *P* < 0.001, resp.). Due to the higher prevalence of women in both groups, a gender analysis was performed for M-SDS and STAY; a significantly higher prevalence of the prevalence of pathological scores for M-SDS, STAY1 and STAY2 was significantly higher CD women than in HC women. Men in the CD group also had a significantly higher pathological score frequency for M-SDS and STAY1 than in the HC (Table 3).

**Figure 2:**
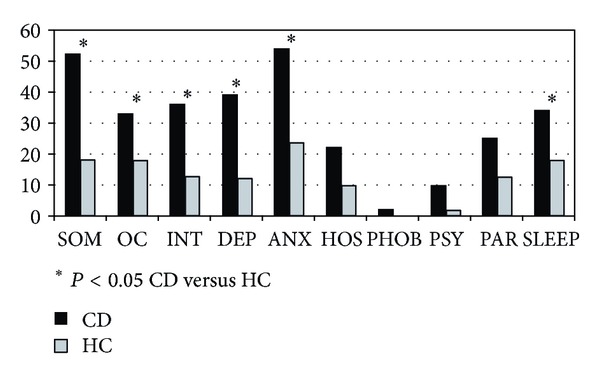
Percentages of pathological SLC-90 subscale scores in celiac disease (CD) patients (black) and healthy controls (HC, grey). Data are expressed as percentages and *P* value (Student's *t*-test for unpaired data).

**Figure 3:**
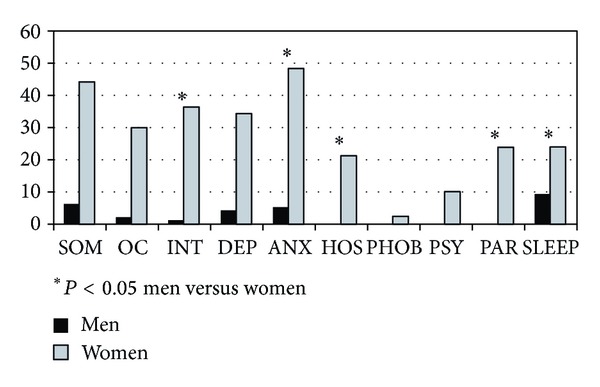
Percentages of pathological SLC-90 subscale scores in celiac disease women and men. ∗ indicate subscales with statistically significant differences (*P* < 0.05) between the two sexes (Student's *t*-test for unpaired data).

Both global and pathological scores from SLC-90 were significantly different between CD and HC (pathological scores: 42% versus 6%, *P* < 0.001). Comparison of single items revealed statistically different pathological values between CD patients and HC for somatization, obsessive-compulsive, interpersonal sensitivity, depression, anxiety, and sleep disorders (Figure 2). Furthermore, when stratifying by gender in the CD group; somatisation, obsessive-compulsive, interpersonal sensitivity, depression, anxiety, and sleep disorders scales were more frequently pathological in women than men (Figure 3).

A multivariate analysis was performed in order to determine the independent effects of CD and gender on eating disorder behaviour. EAT-26 scores, as a surrogate of eating disorder behaviour, were modelled against gender and CD, using M-SDS, STAY1 and STAY2 as covariates. A significant association between EAT-26 score and both CD presence (*P* = 0.02) and feminine gender (*P* = 0.003) without any significant influence of anxiety and depression was found when incorporating M-SDS, STAY1 and STAY2 into the model. In particular, EAT-26 scores were 3.9 units lower in HC than CD after adjusting for depression and anxiety (*B*  −3.9 95% [CI −7.2/−0.5], *P* = 0.03); In the adjusted model, EAT-26 scores were 5 units lower in males than in females (*B*  −5.05 95% [CI −9.5/−0.5], *P* = 0.02). Overall, our results indicate that eating disorder behavior, through EAT-26 scores, was significantly higher in CD patients and in women, whilst there seems to be no significant correlation with the presence of gastrointestinal symptoms.

## 4. Discussion

This study systematically investigates the prevalence and characteristics of eating behaviors in untreated celiac patients and healthy controls through the use of several validated questionnaires. Our findings indicate that the frequency of altered eating behavior is increased in untreated CD when compared with HC. Our data also show that bulimic behavior was found only in CD men. Another corollary, yet relevant datum, is that untreated CD patients, both men and women, report a higher daily carbohydrate intake (that in Southern Italy is mainly due to pasta and bread consumption) in comparison to HC and this finding may support the hypothesis of high gluten intake before the onset of celiac disease in genetically predisposed individuals.

To our knowledge, this study is the first that shows the presence of altered eating behaviors in untreated CD.

The EAT-26 is probably the most used questionnaire to measure symptoms and concerns due to eating disorders. EAT-26 is considered only a first step in the evaluation of a patient followed in case of pathological scores by an interview by qualified professional to assess if the individual meets the criteria for an eating behavior diagnosis. There were no significant differences in the frequency of pathological EAT-26 between CD and HC. However, most of CD patients scored more than 10 items in the EAT-26 scale, not enough to a precise diagnosis but enough to underline a trend toward a strong concern about body and food intake. Regression analyses demonstrated that high scores of EAT-26 in CD do not correlate with presence of gastrointestinal symptoms or with high scores of anxiety and depression scales.

The EDI-2 scale, that is, a self-assessment of symptoms associated with anorexia nervosa and bulimia nervosa showed that CD men were significantly different from HC in the interceptive awareness, that is, the measure of the ability of an individual to discriminate between sensations and feelings and between hunger and satiety. CD women showed higher scores than HC women for pulse thinness, ascetism, perfectionism, and inadequacy. These items suggest the profile of body dissatisfaction, insecurity in one's physical appearance, and strong feelings of inadequacy linked to tendency to the avoidance of sexual relationships, and in general feelings of social insecurity.

The increased frequency of eating behavior in untreated CD might be a consequence of a number of concurrent factors.

Firstly, it might be a feature of the already known altered psychological status of CD [4, 30]. However, in our series the multivariate analysis between EAT-26 and STAY-1 and STAY-2 and M-SDS scales did not disclose any relationship between eating disorders and anxiety and depression indices.

Secondly, an increased frequency of eating disorder could be food-related as carbohydrate intake has been shown to be higher in CD than in HC. In fact, high carbohydrate intakes are related to the presence of gastrointestinal symptoms [30]. Therefore, it is possible that gastrointestinal symptom onset subsequent to food intake (particularly those containing gluten and/or high content of unadsorbable carbohydrates, i.e., FODMAPs) might be a relevant conditioner in altered eating habits in CD [31].

Thirdly, CD patients commonly suffer concomitant functional bowel disorders, as shown by the persistence of gastrointestinal symptoms after initiation of a consolidated strict GFD [32]. However, our data indicate that eating disorders do not correlate with the presence of gastrointestinal symptoms in CD group making this explanation unlikely.

Lastly, it is hypothesized that hormones or other mediators in the blood stream might play a role in the genesis of altered eating behavior in CD [33].

Previous studies related to CD and eating disorders have been limited to only a small number of patients. Currently, through the utilization of a large case control study, we demonstrated that the emotional relationship of untreated celiac patients with food is altered and significantly different from that of a control population. In this study, selection bias was also limited as patients were recruited from a dedicated clinic at the moment of diagnosis, before receiving the prescription to obtain gluten-free food under the coverage of the National Health System.

The main limitation of this study relates to the lack of information on the effect of gluten-free diet on eating disorders (study is in progress). Additionally, collection of information on dietary habits may have been subject to possible recall and observation bias.

In conclusion, the findings of this study further delineate the importance of caregiver vigilance regarding the recognition of eating disorders in celiac patients.

## Figures and Tables

**Table 1:** Basic characteristics of the study population.

	CD	HC	*P*
Number of women	72	68	0.6
Age (means ± SD)	29.2 ± 8.7	30.1 ± 8.2	0.5
BMI (Kg/m^2^)	22.0 ± 3.5	23.30 ± 3.0	**0.005**
% Reporting alcohol intake	15	16	1.0
% Reporting moderate to high physical activity	36	36	0.7
% Reporting gastrointestinal symptoms	**78**	**22**	**0.0001**

Data showed in bold are significantly different (*P* < 0.05) between groups.

CD: celiac disease group; HC: healthy controls group.

**Table:** (a)

EDI-2 women	CD	HC	*P*
Pulse thinness	6.1 ± 4.2	2.6 ± 2.6	**0.001**
Bulimia	1.2 ± 2.0	2.0 ± 2.6	Ns
Dissatisfaction for own body	7.2 ± 5.9	6.1 ± 5.3	Ns
Interpersonal diffidence	4.2 ± 3.4	2.2 ± 1.8	**0.01**
Perfectionism	5.0 ± 2.9	3.1 ± 3.2	**0.03**
Inadequacy	5.2 ± 4.4	2.2 ± 2.2	**0.03**
Interceptive awareness	3.6 ± 3.2	2.2 ± 3.5	**Ns**
Fear of maturity	3.4 ± 3.5	3.7 ± 2.9	**Ns**
Asceticism	5.9 ± 4.5	2.2 ± 2.0	**0.001**
Impulsivity	3.4 ± 4.0	2.3 ± 3.4	**Ns**
Social insecurity	6.7 ± 5.3	3.0 ± 2.6	**0.003**

Data are expressed as means (M) and standard deviation (SD).

Data showed in bold are significantly different (*P* < 0.05) between groups.

Ns: not statistically significant; CD: celiac disease group; HC: Healthy controls group.

**Table:** (b)

EDI-2 men	CD	HC	*P*
Pulse thinness	1.4 ± 1.3	3.3 ± 2.3	Ns
Bulimia	2.2 ± 3.9	1.7 ± 1.2	Ns
Dissatisfaction for own body	4.4 ± 2.8	4.4 ± 2.0	Ns
Interpersonal diffidence	2.8 ± 1.5	1.4 ± 1.7	Ns
Perfectionism	3.4 ± 2.2	1.6 ± 1.3	Ns
Inadequacy	4.0 ± 4.3	2.6 ± 2.3	Ns
Interceptive awareness	4.0 ± 2.9	1.1 ± 1.5	**0.02**
Fear of maturity	4.2 ± 6.3	2.6 ± 1.7	Ns
Ascetisms	3.4 ± 3.5	2.3 ± 2.0	Ns
Impulsivity	2.2 ± 2.3	2.7 ± 2.1	Ns
Social insecurity	4.4 ± 4.8	3.1 ± 3.2	Ns

Data are expressed as means (M) and standard deviation (SD). Data showed in bold are significantly different (*P* < 0.05) between groups.

Ns: not statistically different.

**Table 3:** The percentages of pathological M-SDS, STAI-Y1, and STAI-Y2 scale scores in celiac disease patients (CDP) and healthy controls (HC) according to gender.

	CD	HC	*P*
M-SDS			
Men	**30%**	**0%**	**0.020**
Women	**50%**	**14.7%**	**0.002**
STAI-Y1			
Men	**40%**	**6.3%**	**0.034**
Women	**64.7%**	**23.5%**	**0.001**
STAI-Y2			
Men	40%	12.5%	Ns
Women	**67.6%**	**21.2%**	**0.0001**

Data are expressed as percentages and *P* value (Student's *t*-test for unpaired data).

Data showed in bold are significantly different (*P* < 0.05) between groups.

Ns: not statistically significant; CD: celiac disease group; HC: healthy controls group.
